# Supplementary material for: Association between oral microbiome and seven types of cancers in East Asian population: a two-sample Mendelian randomization analysis
Source: Front Mol Biosci. 2023 Nov 21;10:1327893. doi: 10.3389/fmolb.2023.1327893 (PMC10702768; doi:10.3389/fmolb.2023.1327893)
Supplement: Supplementary file 1 [file DataSheet1.ZIP › Supplementary Materials/MR plots of saliva/saliva═╝/Prostate cancer/pheno.300_to_prostate cancer_forest.pdf]

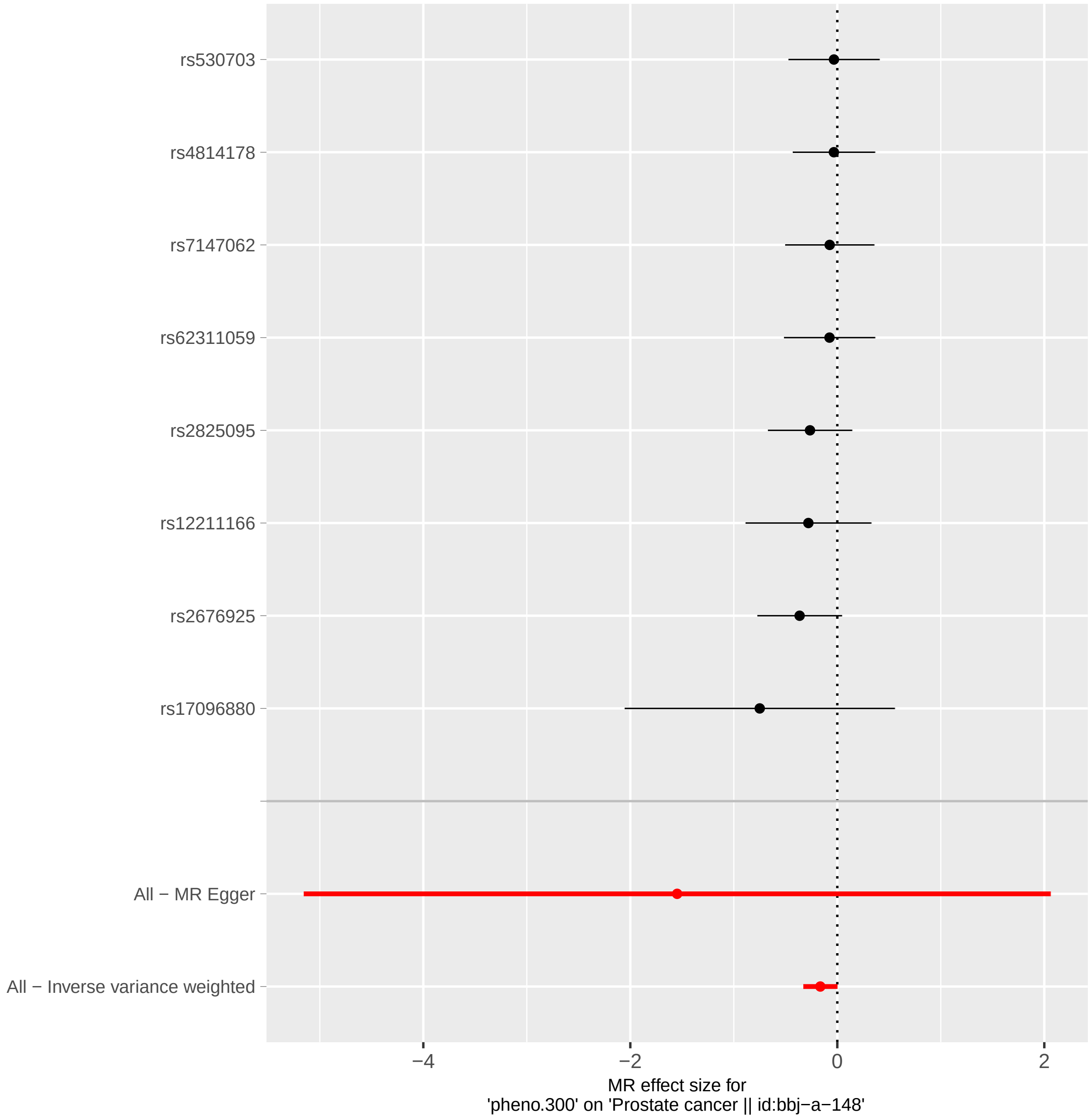

rs530703
rs4814178
rs7147062
rs62311059
rs2825095
rs12211166
rs2676925
rs17096880
All − MR Egger
All − Inverse variance weighted
−4
−2
0
2
MR effect size for
'pheno.300' on 'Prostate cancer || id:bbj−a−148'
